# Actomyosin Interaction: Mechanical and Energetic Properties in Different Nucleotide Binding States

**DOI:** 10.3390/ijms9101927

**Published:** 2008-10-13

**Authors:** Iuliana Aprodu, Alberto Redaelli, Monica Soncini

**Affiliations:** Department of Bioengineering, Politecnico di Milano, Piazza Leonardo da Vinci 32, 20133, Milano, Italy. E-Mails: iuliana.aprodu@polimi.it (I. A.); alberto.redaelli@polimi.it (A. R.)

**Keywords:** Actomyosin, mechanical properties, molecular dynamics

## Abstract

The mechanics of the actomyosin interaction is central in muscle contraction and intracellular trafficking. A better understanding of the events occurring in the actomyosin complex requires the examination of all nucleotide-dependent states and of the energetic features associated with the dynamics of the cross-bridge cycle. The aim of the present study is to estimate the interaction strength between myosin in nucleotide-free, ATP, ADP·Pi and ADP states and actin monomer. The molecular models of the complexes were constructed based on cryo-electron microscopy maps and the interaction properties were estimated by means of a molecular dynamics approach, which simulate the unbinding of the complex applying a virtual spring to the core of myosin protein. Our results suggest that during an ATP hydrolysis cycle the affinity of myosin for actin is modulated by the presence and nature of the nucleotide in the active site of the myosin motor domain. When performing unbinding simulations with a pulling rate of 0.001 nm/ps, the maximum pulling force applied to the myosin during the experiment is about 1nN. Under these conditions the interaction force between myosin and actin monomer decreases from 0.83 nN in the nucleotide-free state to 0.27 nN in the ATP state, and increases to 0.60 nN after ATP hydrolysis and Pi release from the complex (ADP state).

## 1. Introduction

Myosins are molecular motors which interact with actin filaments and employ energy from ATP hydrolysis to provide conformational changes, which generate force. Three-dimensional structures of both myosin and actin have been recently solved by high resolution X-ray crystallography. The crystallographic structures of chicken skeletal myosin subfragment-1 (S1) [1], Dictyostelium discoideum myosin II truncated head [2–7], vertebrate smooth muscle myosin motor domain [8], and scallop myosin S1 [9–13] were obtained and deposited in the RCSB Protein Data Bank. The actin molecule, which is the myosin substrate, has been solved too; atomic models of a single rabbit actin monomer [14, 15] complexed with gelsolin [16, 17] or DNase 1 [18] are available. However, all crystallographic models of myosin in different nucleotide binding states were obtained in absence of actin, since a co-crystallization seems still impossible to achieve. Nevertheless, a first approximation of the atomic model of the actomyosin complex can be obtained by fitting the atomic structures of actin and myosin motor domain into three-dimensional cryoelectron microscopy maps of decorated actin [19].

Experimental studies of light microscopy and single molecule manipulation, coupled with structural biology techniques (X-ray and electron crystallography, NMR and cryoelectron microscopy) show that the affinity of myosin for actin is modulated by the presence and the nature of the myosin nucleotide binding site. When the nucleotide is absent, the myosin head binds tightly to the actin filament to form the “rigor” complex. The binding of the ATP molecule causes the rapid dissociation of the actomyosin complex; the myosin head undergoes reversible closure of the ATP binding pocket and converter domain rotation [20]. When the ATP hydrolyses and the stable ADP·Pi state is formed, the myosin weakly binds the actin filament; such coupling causes the Pi release and the subsequent power stroke. At the end of the process, the ADP is also released and the rigor conformation is recovered. Most of the structural details about each state come from high resolution structures of Dictyostelium discoideum myosin with different ligands and from kinetic analysis of different mutants. According to these studies, the myosin motor domain adopts different nucleotide-dependent conformations: the open state when the active site is empty or bound with Mg·ATP [21], Mg·ATP analogues (e.g., Mg·ATP*γ*S and Mg·AMP·PNP) and Mg·ADP [6], and the closed state when the active site is complexed with analogues of the ATP, such as Mg·ADP·AlF_4_^-^ [2] and Mg·ADP·VO_4_ [4].

In addition to experimental observations, a detailed picture of the myosin conformational changes during an ATP hydrolysis cycle results from the computational approach, which complements structural and biochemical studies. The mechanism of ATP hydrolysis in myosin II was qualitatively investigated by both molecular dynamics (MD) and quantum mechanics, combined with molecular mechanics calculations, for two different states (ATP and ADP·Pi) of the motor domain [22, 23]. MD simulations were also performed by Kawakubo *et al.* [24] to model the propagation of the ATP hydrolysis energy, and by Lawson *et al.* [25] to study the structural mechanism of the back door phosphate release. Liu *et al.* [26] used the MD approach to explore the interactions between myosin subdomains and actin. In this latter study, the authors considered a rigor conformation model composed of one myosin head and two actin monomers. Two different myosins (wild-type and R405Q mutant) were considered and in both cases the results showed that the overall interaction between the proteins within the complex is mainly due to electrostatic interactions.

**Figure 1.**
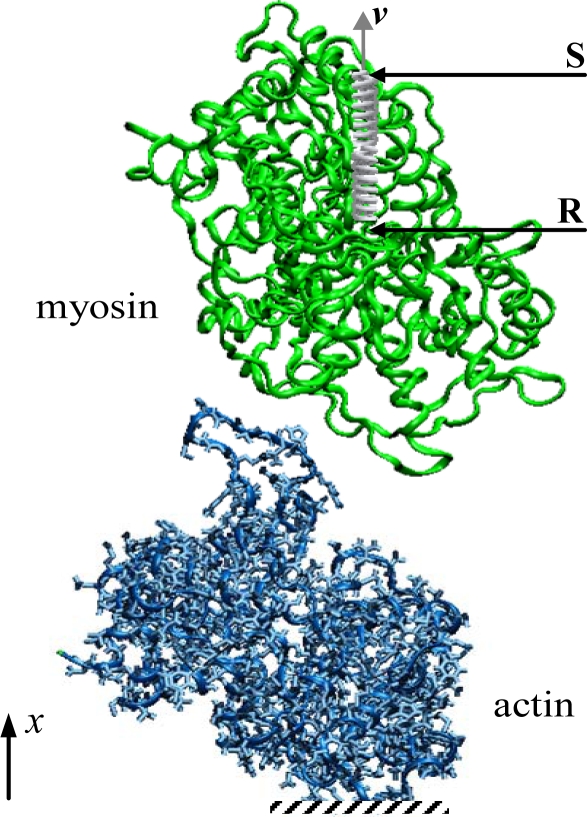
Simulation set-up for the evaluation of the interaction properties between myosin motor domain and actin monomer. The actin monomer was fixed, while the myosin motor domain was moved by means of a virtual spring. One end of the spring (R) was attached to the center of mass of the motor protein, while the free end of the spring (S) was moved at constant velocity (ν) in a direction (*x*) perpendicular to the longitudinal axis of the actin filament.

A complete understanding of the actomyosin interaction requires information about all the nucleotide-dependent states that the actomyosin complex takes on during a cycle of ATP hydrolysis. In order to address this issue, we propose a new approach based on MD to evaluate the interaction properties of the actomyosin complex. Given the time scale involved (i.e., 1–10 ms for each ATP cycle), the continuous attachment and detachment mechanism cannot be simulated; consequently in the present study four different states (nucleotide-free, ATP, ADP·Pi and ADP) of the actomyosin complex were considered. The actomyosin models consist of one molecule of each type, assuming that within the complex the interaction properties are mainly given by the myosin interfacing with one actin monomer having a particular orientation in the filament. The investigation of the energetic and mechanical properties of the actomyosin complex was carried out on equilibrated complexes. The interaction energy of the complex was estimated by means of Steered Molecular Dynamic (SMD) simulations [27, 28]; specifically, the actin monomer was fixed, while the myosin motor domain was moved closer and further away using a spring (Figure 1). The interaction force and binding stiffness were calculated on the basis of energy curves.

The appeal of the method adopted in the present work lies in the fact that it allows investigations of the energetic and mechanical properties at the molecular scale; moreover it can accurately reproduce the *in-vitro* experimental set-up used to carry out unbinding measurements, providing information that is not accessible via experimental tests.

## 2. Results and Discussion

### 2.1. Analysis of the Equilibrated Structures

The four actomyosin complexes in different nucleotide states (nucleotide-free, ATP, ADP·Pi and ADP) were minimized, solvated and equilibrated at a temperature resembling the experimental one. After about 600 ps of equilibration dynamics, the root mean square deviation (RMSD) of the Cα atom position with respect to the starting structures reached the stable value of 0.57±0.03 nm (mean±SD), 0.26±0.02 nm, 0.83±0.08 and 0.24±0.02 nm, respectively in the case of nucleotide-free, ATP, ADP·Pi and ADP complexes.

Table 1 provides the conformational details of the complexes as calculated by using the Procheck software [29]; the individual stereochemical quality of the structures is meant as a measure of the reliability of the equilibrated models. The fully, additionally and generously allowed regions indicate the permitted backbone conformations for psi/phi angle pairs for each residue of the protein. The high percentage of residues belonging to the allowed regions supports the good reliability of the obtained results.

### 2.2. Analysis of the Interaction Properties of the Actomyosin Complex

In the literature, experimental investigations of the interaction forces between actin and myosin at the single molecule level are usually done using two different types of set-ups. The former consists in the actin filament attached to a probe held by a force transducer and presented to myosin molecules bound to a substrate [30–32]. The latter assumes that actin is bound to a surface, while the motor protein, attached to plastic or glass beads held by force transducers is presented to the counterpart filament [33]. The most of the experimental apparatuses frequently used as force transducers are atomic force microscopes (AFM), and optical tweezers; the proteins’ interactions are estimated using different types of photodiode detectors.

In the present work we used a MD approach to investigate the characteristics of the forced unbinding of myosin motor domain from an actin monomer. This study was motivated by experimental measurements of the unbinding process [33]; both in single-molecule experiments and in computer simulations, the proteins’ unbinding is obtained by applying external forces that are large enough to overcome the potential energy barriers. A correct estimation of the interaction properties of the actomyosin complex depends on the choice of the appropriate parameters for the SMD simulations. Therefore, a preliminary sensitivity analysis was performed to define the optimal pulling parameters to be used for the simulation of the actomyosin complex unbinding.

### 2.3. Influence of the Pulling Rate

**Figure 2.**
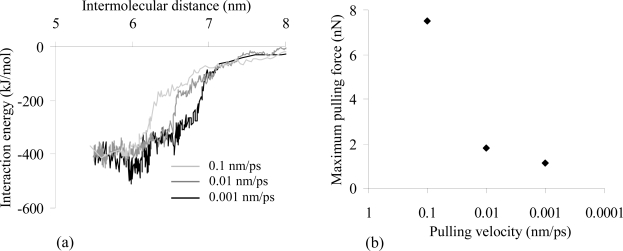
**(a)** Interaction energy vs. intermolecular distance for three SMD tests performed with a spring constant of 3000 kJ mol^−1^ nm^−2^ and different pulling rates (ν = 0.1 nm/ps in light grey, ν = 0.01 nm/ps in grey and ν = 0.001 nm/ps in black); **(b)** Maximum pulling force vs. pulling rate.

The equilibrated structure of the actomyosin complex in ADP·Pi state was used to evaluate the influence of the pulling rates (ν) on the molecular behaviour of the system during the forced unbinding simulation. Three pulling rates – 0.1 nm/ps, 0.01 nm/ps and 0.001 nm/ps – were tested using a spring constant of 3000 kJ mol^−1^ nm^−2^. The obtained interaction energy vs. intermolecular distance profiles (Figure 2a) are qualitatively similar in the three cases; the minimum interaction energy decreases when the value of ν is lowered. Moreover, at higher pulling rates (0.1 and 0.01 nm/ps), a plateau of minimum interaction energy was reached when the two proteins were approached; at a pulling rate of 0.001 nm/ps, a minimum value of the interaction energy was obtained at an intermolecular distance of about 6 nm.

In agreement with the results of the experimental unbinding forces of different biological complexes [34, 35], our results (Figure 2b) suggest that the lower the pulling velocities are, the smaller the forces needed to move the myosin apart from the actin become; decreasing the velocity from 0.1 to 0.001 nm/ps reduces the value of the maximum pulling force from 7492 pN to 1023 pN. The force required to unbind biological complexes strongly depends on the pulling velocity. Merkel *et al.* [34] studied both avidin-biotin and streptavidin-biotin unbinding behaviour by means of dynamic force microscopy. Different loading rates were tested over six orders of magnitude (from 0.05 to 60000 pN/s) and the unbinding force increased from 5 to 170 pN. In addition, atomic force microscopy experiments on P-selectin revealed that the force required to unbind the ligand-receptor complex has a logarithmic dependence on the pulling velocity [35].

Even if the lowest maximum pulling force (1023 pN) obtained by performing SMD simulation with a pulling rate of 0.001 nm/ps is still one order of magnitude higher than the experimental one [34], it is weak enough and changes very slowly in time to induce minor distortions in the molecular structures during pulling experiment simulation.

Generally, SMD simulations meant to induce conformational changes or proteins’ unfolding are very sensitive to the pulling rate [27]; when high pulling rates are being used, unphysical trajectories are yielded. Unlike unfolding SMD simulations, the set-up adopted for the characterization of the actomyosin complex allows a proper estimation of proteins’ behaviour during unbinding as long as proteins distortions are limited [27].

The pulling velocity used in the SMD simulations is inversely related to the unbinding time; at low velocities the molecular system has more time to thermally equilibrate and consequently oscillations were recorded due to the continuous rearrangements of the proteins (Figure 2b); this phenomenon resembles the biological behaviour of the proteins.

Moreover, since we deal with globular-shaped proteins, the large size of the solvated models (about 127000 atoms) imposes a pulling rate of 0.001 nm/ps, comparable with the one adopted in previous computational studies on different complexes, which successfully matched the experimental data [36, 37] but still higher than the pulling rates used regularly in AFM experiments [38], which are in the nanometres per milliseconds range. Indeed, considering that MD simulations were performed in parallelization conditions (Intel Core2 CPU 6300 1.86 GHz), the CPU time was 0.2 ns per day. Decreasing the pulling rate of one order of magnitude (from 0.001 nm/ps to 0.0001 nm/ps) would increase the total CPU time required to evaluate the interaction properties of each state of the actomyosin complex, from 25 days up to more than 250 days due to the high oscillations of the system.

Taking into account the abovementioned observations, the lowest tested pulling velocity (0.001 nm/ps) was used to perform the SMD simulations for the analysis of the different nucleotide states of the actomyosin complex.

### 2.4. Influence of the Spring Constant

The effect of the spring constant on the mechanical behaviour of myosin when it is moved apart from the actin monomer was evaluated through SMD simulations at a constant rate of 0.001 nm/ps with three different *k* values, 300, 3000 and 10000 kJ mol^−1^ nm^−2^.

**Figure 3.**
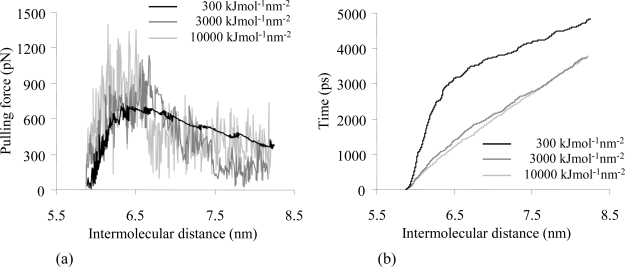
**(a)** Pulling force vs. intermolecular distance and **(b)** simulation time vs. intermolecular distance profiles for SMD performed with a pulling rate of 0.001 nm/ps and different spring constants (*k* = 300 kJ mol^−1^ nm^−2^ in black, *k* = 3000 kJ mol^−1^ nm^−2^in grey and *k* = 10000 kJ mol^−1^ nm^−2^ in light grey).

When a stiff spring is used to pull apart the myosin from the actin monomer, large amplitude fluctuations of the pulling force profile can be observed, and some filtering might be considered in order to reveal the local events. Softer springs exert a less restricted action on the molecular system; in this case local displacements are detected (Figure 3a). Our results in terms of force oscillations agree with the observations of Lorenzo and Bisch [39]. When thermodynamic systems are subjected to external oscillatory forces, the amplitude of the displacement (δ*_x_*) due to the thermal energy fluctuation can be estimated by means of the expression: δ*_x_*= *(k_B_T/k)^1/2^*, where *k_B_* is the constant of Boltzmann and *T* is the absolute temperature (*T* = 300K). Since the amplitude of the force oscillations is δ*F* = *-k* δ*_x_*, then the following relationship is obtained: δ*F* = *-(k_B_Tk)^1/2^* [39]. The results of the SMD simulations performed with different *k* (Figure 3a) comply with these theoretical models. For instance, in case of *k*=3000 kJ mol^−1^ nm^−2^ (4.98 N/m), the mean value of the amplitude of the force oscillations measured during SMD simulation is about 180 pN (Figure 3a), while the estimated value obtained by theoretical models is equal to 145 pN.

The simulation time required to pull the myosin motor domain 2 nm apart from actin was 4.8 ns when using *k* =300 kJ mol^−1^ nm^−2^, while in case of higher spring constants (*k* = 3000 kJ mol^−1^ nm^−2^ and *k* = 10000 kJ mol^−1^ nm^−2^) the simulation time decreased of about 27% (Figure 3b).

The best compromise in terms of limiting the amplitude of oscillations introduced in the force values and reducing the simulation time, was obtained for a spring constant of 3000 kJ mol^−1^ nm^−2^.

### 2.5. Evaluation of the Interaction Forces Characterising the Actomyosin Complexes

**Figure 4.**
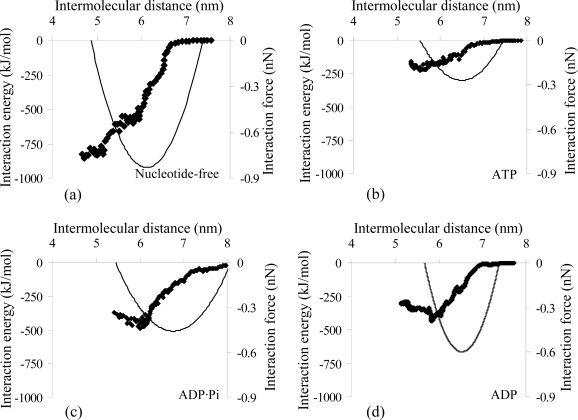
Profiles of the interaction energy (dots) and interaction forces (lines) as function of intermolecular distance of the actomyosin complex in different nucleotide-dependent conformations; nucleotide-free (a), ATP (b), ADP·Pi (c), ADP (d).

Using the optimal pulling parameters defined previously (ν = 0.001 nm/ps, *k* = 3000 kJ mol^−1^ nm^−2^), the interaction properties of the actomyosin complex in different nucleotide-dependent conformations were estimated *via* SMD simulations, starting from the equilibrated model of each state. We monitored the intermolecular distance and the interaction energy between the two proteins of the actomyosin complex and we evaluated the interaction forces and binding stiffness (Figure 4 and Table 2) by means of Eq. 3 and Eq. 4 (see Experimental Section).

Our results predict that the conformational changes occurring in the motor domain of the myosin during a cycle of ATP hydrolysis markedly influence the interaction forces and the energy profiles of the actomyosin complex.

**Figure 5.**
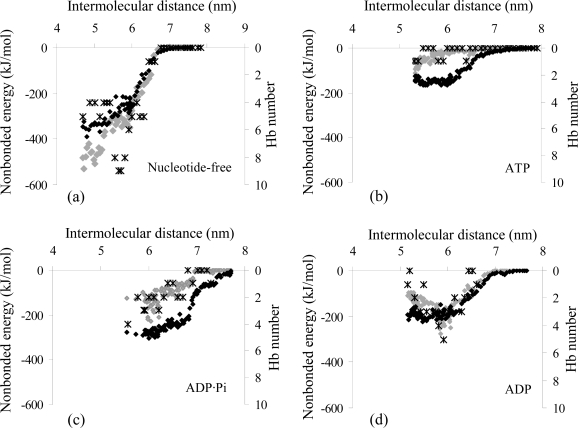
Profiles of Coulomb energy term (grey dots), van der Waals energy term (black dots) and Hb number connecting the actomyosin complex (crosses) as a function of intermolecular distance, in different nucleotide-dependent conformations. nucleotide-free (a), ATP (b), ADP·Pi (c), ADP (d).

The interaction force vs. intermolecular distance curves (Figure 4) are approximations, valid only around the minimum value of the forces, but are not representative for systems behaviour in general. Nevertheless the maximum interaction forces estimated for each nucleotide-dependent actomyosin complex (*F_max_*, Table 2) are a quantitative estimation, which reflect the coupling behaviour shown by the interaction energy profiles in Figure 4 and by the non-covalent interactions (van der Waals and Coulomb energy terms, Figure 5) acting between the actin monomer and the myosin in the complex. The rigor conformation stands out for a depth falling of the interaction energy curve when myosin motor domain approaches the actin monomer, while the energy curves calculated for ATP, ADP·Pi and ADP states have shallow minima when the proteins are moved toward. This behaviour is representative of a weak coupling between two proteins and enables their relative motility.

In all the studied cases the non-bonded energy term lowers with the decrease of the intermolecular distance until the minimum interaction energy is achieved. As shown in Figure 5, at the equilibrium distance (intermolecular distance corresponding to the energy minimum) the contribution of the electrostatic attraction to the net interaction energy is dominant when the actomyosin complex is in the nucleotide-free and ADP states (62% and 65%, respectively), in agreement with Liu *et al.* [26]; on the other hand, in the ATP and ADP·Pi states the van der Waals term dominates (75% and 55%, respectively). Consequently, the Pi release from the complex is coupled with a decrease of the van der Waals contribution (from 271 to 147 kJ/mol) and a growth of electrostatic contribution (from 219 to 276 kJ/mol) to the total interaction energy term.

In addition, the number of the inter-chain hydrogen bonds (Hb) vs. intermolecular distance (Figure 5) is in agreement with the affinities between the proteins during a cycle of ATP hydrolysis (Figure 4); in fact complexes characterized by higher interaction forces are connected by a higher number of Hb. A detailed analysis of Hb connecting the interfaces of the equilibrated actomyosin complexes in different nucleotide-dependent states gives also valuable qualitative indications about the affinities at the atomic level within the complex during an ATP hydrolysis cycle. The hydrogen bridging of myosin (M) and actin (A) occurring when the nucleotide-binding cleft of the motor protein is open, is mainly due to the following Hb: Lys448 (M) - Glu93 (A), Lys550 (M) - Met47 (A), Tyr552 (M) - Glu93 (A), Asp553 (M) - Lys50 (A). When the nucleotide-binding cleft is closed, the proteins’ interfaces are mainly bridged by 5 Hb: Lys530 (M) - Met45 (A), Lys530 (M) - Glu58 (A), Arg546 (M) - Glu100 (A), Phe547 (M) - Arg96 (A), Lys549 (M) - Tyr92 (A). In both cases the hydrogen bridging of the actomyosin complex involves the residues of the motor protein’s primary binding site. The low number of Hb connecting the complex indicates that the electrostatic and van der Waals terms are primarily responsible for the actomyosin interaction.

Our findings are in agreement with the experimental observations by Rayment *et al.* [20]. The *in-vitro* motility assay, combined with X-ray crystallography and molecular biology techniques, shows that myosin in the rigor state (nucleotide-free) is usually locked tightly to the actin filament (interaction force F_max_=0.83 nN, Table 2; electrostatic attraction is dominant, Figure 5), while the affinity between the two proteins decreases when the ATP nucleotide is bound to the large cleft of the myosin head (F_max_=0.27 nN, Table 2; van der Waals contribution is dominant, Figure 5); after ATP hydrolysis (F_max_=0.46 nN, Table 2; van der Waals contribution is dominant, Figure 5), the release of the Pi from the complex triggers the power stroke and generates the myosin(ADP)-actin state (F_max_=0.60 nN, Table 2; electrostatic attraction is dominant, Figure 5), which is the intermediate complex that leads to the rigor state after the ADP release [20].

The interaction strength modulation is supported by the conformational changes of the myosin motor domain. In strongly bound states, the actin-binding cleft is closed and 50K domain of the myosin interact with actin monomer, while in weakly bound states the actin-binding cleft is open and only the lower 50K domain establish weak stereospecific interactions with the actin [40]. These conformational changes of the myosin determine large variations of the interaction surface of the actomyosin complex. The interaction surface of both myosin motor domain and actin monomer varies with the type of the adenine nucleotide attached to the nucleotide binding pocket of the motor protein. As suggested by Holmes *et al.* [40], our results (Table 2) indicate that the interaction surface (*S*) of the actomyosin complex halves when passing from rigor state (833.32 Å^2^) to weakly bound state (402.08 Å^2^). Concerning the nucleotide-free complex, the interaction surface is comparable to the one estimated by Liu *et al.* [26] when performing MD simulations on the complex with two actin monomers. This similarity gives indications about the reduced contacts between myosin and the adjacent actin monomer.

With regard to experimental data from literature to be used for comparison, most of the investigations on the single actomyosin complex are focused on the displacement and the force generated during a cycle of ATP hydrolysis [32,41]. Other experiments performed at single molecule level give indication about the value of the unbinding force [30–33]. Nakajima *et al.* [33] monitored the interaction events occurring on single myosin head and actin by means of scanning force microscopy and measured an unbinding force of 14.8 pN [33], in good agreement with the value obtained by Nishizaka *et al.* [30, 31] via optical tweezers. These values are lower than the ones obtained in the present study by means of SMD. This discrepancy is possibly due to the differences in the set-up adopted in the MD approach and in the experimental procedure. In SMD simulations the myosin motor domain was pulled through an unbinding direction perpendicular to the longitudinal axes of the actin filament while in experimental set-ups the direction of the imposed loads varied within a wide range. The peeling procedure used in Nakashima’s and Nishizakas’ experiments [30, 31, 33] likely cause a distortion of the actomyosin interface altering their conformational match. Small deformations of proteins’ structures could reduce significantly the binding interface and consequently the binding strength.

Moreover, as shown previously, the pulling force and consequently the interaction force highly depend on the pulling velocity. Since the unbinding force is smaller when the external load is applied slowly, we may expect a slight further decrease of the interaction force when performing SMD simulations with pulling rates lower than 0.001 nm/ps.

A significant difference between MD simulations [42] and measurements by AFM experiments [43] was observed for the avidin-biotin molecular system. As pointed out by Izrailev *et al.* [42], the different values of the interaction forces arise from the different time scales of the simulated and experimental tests. In the AFM experiment performed by Florin *et al.* [43] the unbinding time was ~1 ms and the measured force was about 160 pN, while in the case of MD [42] the simulation time was about 1 ns and the obtained unbinding forces were significantly larger (450 pN). In the case of AFM and similar manipulation techniques, the unbinding time is usually of the order of a millisecond, long enough to allow the proteins to thermally equilibrate and to overcome thermal barriers. In the case of molecular systems of the same size of the one considered in this study, MD simulations can cover only a timescale of the order of nanoseconds; hence, the unbinding event occurs in a dissipative regime by applying large forces, which are able to overcome relevant energy barriers and induce a sufficiently rapid dissociation [42].

## 3. Experimental Section

### 3.1. Molecular Models

The X-ray crystallographic models of both open and closed forms of the myosin motor domain and of the actin monomer were obtained from the Brookhaven Protein Data Bank. Four different nucleotide-dependent binding states (nucleotide-free, ATP, ADP·Pi and ADP) of the actomyosin complex were built. With regard to myosin, we used *Dictyostelium discoideum* data; the nucleotide-free state (when the motor protein is tightly locked to actin filament) was modelled starting from 1FMV.pdb [21], while the weak binding states (ATP, ADP) were obtained starting from 1FMW.pdb [21] and 1MND.pdb [2], respectively. The myosin(ADP·Pi) complex was built starting from the equilibrated model of 1FMW.pdb. In order to model actin, we used 1ATN.pdb [18], which is an atomic model of the complex between rabbit skeletal muscle actin and bovine pancreatic DNase I. The myosin models were docked on actin monomer using 1O1B.pdb [19] as template configuration. In particular, the Least Squares Fitting (LSQ-fitting) algorithm was used to set the relative positions of the proteins and to properly fit the interfaces.

Each complex was prepared for the MD simulations by removing all the water molecules embedded in the structures and all the chemical compounds (chloride, magnesium and tetrafluoroaluminate ions) added during the crystallization procedure for the stabilization of the proteins and not physiologically present in biological systems.

### 3.2. Numerical Set-up

Energy minimizations and MD calculations were performed by means of the Gromacs 3.3 software, using the GROMOS96 43a1 force field. All simulations were carried out in parallelization on an Intel(R) Core(TM)2 CPU 6300 1.86 GHz processor-based Linux running machine.

To ensure that the investigated actomyosin complexes were free from strongly repulsive non-bonded contacts or geometric distortions inconsistent with the potential energy function, a preliminary in vacuum energy minimization of each system was performed. The energy minimization was carried out using a sequence of two algorithms (steepest descent and limited-memory Broyden-Fletcher-Goldfarb-Shanno (L-bfgs).

The actomyosin models were then solvated in rectangular boxes (17 nm x 6 nm x 13 nm) with about 38460 Single Point Charge (SPC) water molecules, bringing the total size of the systems to about 126764 atoms each. In order to reduce the computational costs associated with water molecules calculations, the box dimensions were chosen as small as possible to get a water shell surrounding the protein complex with a thickness of at least 1 nm (except for *x* direction, Figure 1). The box dimension in the *x* direction was instead set large enough to perform SMD simulations. The total charge of the solvated protein complexes was neutralised by adding Na^+^ counter ions. Each system was then prepared for the MD steps by performing a further energy minimization.

In order to gradually increase the system temperature from 0 to 300K, proteins and solvent were separately coupled to a Berendsen thermostat for 100 ps, and a time constant of 0.1 ps was used to produce a weak coupling between temperature and atom velocities [44]. To finally achieve the starting models for the SMD simulations, each system was further equilibrated for 1 ns.

MD simulations were performed using the leap-frog integration with a time-step of 2 fs and the atomic coordinates of the whole system were recorded every ps. The van der Waals interactions were cut off beyond 1 nm. The Particle-Mesh-Ewald (PME) method [45] was used to allow for the calculation of the long-range electrostatic interactions using a Fourier spacing of 0.12 nm and a fourth order interpolation.

The investigation of the interaction forces that characterise the actomyosin complex in different nucleotide-dependent states was carried out by performing SMD simulations that started from the equilibrated model of each complex. During the simulation the actin monomer was fixed by blocking the spatial coordinates of the Cα atoms, while the myosin motor domain was moved closer and further by means of a virtual spring. One end of the spring (R) was attached to the center of mass of the myosin molecule (Figure 1), while the other end (S) was moved along the *x* direction at a constant rate *v* defined as:

(1) $$\nu =\frac{dx_{S}(t)}{dt}$$

where *x_S_* (*t*) represents the coordinate in *x* direction of the free end of the spring as a function of time, *t*.

The pulling force acting on the myosin motor domain was calculated as:

(2) $$F_{P}(t)=k(x_{S}(t)-x_{R}(t))$$

where *x_R_* (*t*) is the coordinate of the end of the spring attached to the myosin center of mass (R), and *k* is the value of the spring constant.

In order to properly set the parameters of the SMD, the final equilibrated structure of the actomyosin complex in the ADP·Pi state was used to perform an analysis of sensitivity to the pulling rate (*v*) and to the spring elastic constant (*k*). Concerning the pulling rate tests, the *k* value was set at 3000 kJ mol^−1^ nm^−2^, while the myosin was moved with velocities of 0.1, 0.01 and 0.001 nm/ps. Three other SMD simulations with a *v* of 0.001 nm/ps and a *k* of 300, 3000 and 10000 kJ mol^−1^ nm^−2^ were performed to test the influence of the spring constant.

For each nucleotide-dependent actomyosin complex, the interaction energy (*V_int_*) was calculated as the sum of the Coulomb and the van der Waals energy terms and sampled regularly together with the intermolecular distance (*r*) during the simulations. The intermolecular distance was calculated as the distance between the centers of mass of the myosin and actin monomer.

The interaction energy vs. intermolecular distance (*r*) data points were fitted using a third order polynomial function. Consequently, the interaction force (*F_int_*) was estimated as the first derivative of the obtained curve:

(3) $$F_{\text{int}}=-\frac{dV_{\text{int}}}{dr}$$

The binding stiffness (*k_int_*) was evaluated as the second order derivative of the interaction energy with respect to the intermolecular distance, calculated at the energy minimum distance (*r_Vmin_*):

(4) $$k_{\text{int}}=\frac{d^{2}V_{\text{int}}}{dr^{2}}{|}_{r=r_{V\;\text{min}}}$$

## 4. Conclusions

In conclusion, the present study provides evidences for the modulation of actomyosin interaction strength during a cycle of ATP hydrolysis. Four different nucleotide binding states (nucleotide-free state in which the nucleotide binding cleft is open, ATP, ADP·Pi and ADP states in which the cleft is closed) of the actomyosin complex were constructed and characterised by means of SMD simulations.

It is well known that myosin interacts mainly with the subdomain 1 of the actin monomer and may establish slight contacts with the subdomain 2 of the adjacent monomer of the actin filament [46, 47]. Cryoelectron microscopy maps and image analysis of the rigor actomyosin complex indicate that there are four distinct regions of the motor protein interacting with its substrate [48]. The myosin’s primary binding site (Pro529-His558 and Gln 649-Lys659), together with its flanking loops Tyr626-Gln647 and Pro404-Lys415 interact with the same actin monomer. The contact with the adjacent substrate monomer is negligible since involves only five residue of the actin (His40-Gly42 and Glu99 -Glu100) which are well positioned to interact mainly with the secondary binding site (Lys567-His578) of myosin [48]. Taking into account the limited interaction between myosin and adjacent actin monomer and for sake of computing time, in the actomyosin molecular models we consider only the actin monomer which mainly interact with the motor protein.

A preliminary sensitivity analysis was performed to define the optimal pulling parameters to be used for the unbinding of the complexes. The results suggest that lower pulling rates are associated with lower pulling forces and energy minima, longer unbinding times and higher oscillating motion of the pulled protein. With regard to the spring constant stiffness tests, our results indicate that a *k* of 3000 kJ mol^−1^ nm^−2^ is a good compromise between system stability and CPU time required to simulate proteins’ unbinding.

The conformational changes occurring in the motor domain of the myosin during a cycle of ATP hydrolysis strongly influence the interaction forces of the actomyosin complex. When performing SMD simulations with a pulling rate of 0.001 nm/ps the interaction force between myosin and actin monomer decreases from 0.83 nN in the nucleotide free state to 0.27 nN in the ATP state, and increases to 0.60 nN after ATP hydrolysis and Pi release from the complex (ADP state). A detailed check of the non-covalent forces acting between proteins within the complex indicated the contribution of the van der Waals and Coulomb energy terms. In the rigor state, the non-bonded energy contribution is mainly due to electrostatic interactions; the closure of the nucleotide binding pocket influences the interaction energy profile: the contribution of the van de Waals forces to the total interaction energy increases to 75%.

The major limitation of the method used in the present work to characterise the actomyosin complex lies in the high velocity used to unbind the two proteins. The rapidly growing computer power together with the progresses made in the last years in developing new algorithms and methods in the field of molecular modelling, will help bridge the gap in time scales and system sizes between computer simulations and experiments.

## Figures and Tables

**Table 1.** Conformational details of the actomyosin complex geometry.

	Actomyosin(free)	Actomyosin(ATP)	Actomyosin(ADP·Pi)	Actomyosin(ADP)
Fully allowed regions (%)	62.63	66.84	64.30	64.31
Additionally allowed regions (%)	30.06	27.11	29.46	28.47
Generously allowed regions (%)	4.54	3.97	4.52	5.24
Disallowed regions (%)	2.77	2.08	1.72	1.98

**Table 2.** Interaction surface (*S*), maximum pulling force (*F_Pmax_*), minimum interaction energy (*V_min_*), corresponding intermolecular distance *(r_Vmin_)*, maximum interaction force (*F_max_*), and elastic constant of the interaction (*k_int_*) evaluated at *r_Vmin_*.

	Actomyosin (free)	Actomyosin (ATP)	Actomyosin (ADP·Pi)	Actomyosin (ADP)
*S* (Å2)	833.32	402.08	543.82	616.09
*FPmax* (nN)	1.79	1.16	1.02	1.64
*Vmin* (kJ/mol)	−857.03	−224.49	−490.67	−422.56
*rVmin* (nm)	4.72	5.55	5.96	5.86
*Fmax* (nN)	0.83	0.27	0.46	0.60
*kint* (pN/nm)	6583.84	727.68	400.39	1139.81
